# Effects of Non-Nutritive Sweeteners and Sweet Taste Exposure on Weight Management, Biomarkers of Health and Sweet Taste Preference—A Review of the Evidence from Recent European Consortia Studies

**DOI:** 10.3390/nu18111647

**Published:** 2026-05-22

**Authors:** Eva Marija Čad, Katherine M. Appleton, Ellen E. Blaak, Clarissa Dakin, Kees de Graaf, Graham Finlayson, Ciarán G. Forde, Jason C. G. Halford, Louise Kjølbæk, Monica Mars, J. Alfredo Martinez, Santiago Navas-Carretero, Anne Raben, Corey Scott, Joanne A. Harrold

**Affiliations:** 1 Department of Food Science and Technology, Biotechnical Faculty, University of Ljubljana, 1000 Ljubljana, Slovenia; 2 Division of Human Nutrition and Health, Wageningen University and Research, 6708 WG Wageningen, The Netherlands; 3 Department of Psychology, Faculty of Science and Technology, Bournemouth University, Poole BH12 5BB, UK; 4 Department of Human Biology, Maastricht University Medical Center, 6229 HX Maastricht, The Netherlands; 5 School of Psychology, University of Leeds, Leeds LS2 9JT, UK; 6 Department of Psychology, The University of Liverpool, Liverpool L69 7ZX, UK; 7 Department of Nutrition, Exercise and Sports, University of Copenhagen, 1165 København, Denmark; 8 Centro de Investigación Biomédica en Red, Fisiopatología de la Obesidad y Nutrición, Instituto de Salud Carlos III, 28029 Madrid, Spain; 9 IMDEA Food Institute, 28049 Madrid, Spain; 10 Endocrinology and Nutrition Center, Universidad de Valladolid, 47003 Valladolid, Spain; 11 Center for Nutrition Research, University of Navarra, 31008 Pamplona, Spain; 12 Navarra Institute for Health Research, 31008 Pamplona, Spain; 13 Cargill R&D Center, B-1800 Vilvoorde, Belgium

**Keywords:** non-nutritive sweeteners, sugar, weight management, glycemia, sweet taste, microbiota, appetite

## Abstract

Non-nutritive sweeteners (NNSs) are consumed to reduce intake by providing a sweet taste with little to no energy. Despite regulatory approval and extensive use, uncertainty remains about their long-term role in weight management and health, and about whether exposure to sweet taste itself, independent of energy, influences these outcomes. This narrative review synthesizes evidence from three recent European consortia: SWEET, SWITCH and Sweet Tooth, which together provide complementary data from acute, short- and long-term randomized controlled trials. The studies examined the effects of NNSs and dietary sweet taste exposure on body weight, health-related biomarkers, sweet taste preference, and eating behavior. Across studies, replacing sugars with NNSs appeared to support weight loss maintenance, while NNS consumption and dietary sweet taste exposure showed no adverse changes in body weight, glucoregulatory and endocrine biomarkers, cardiometabolic risk factors, gut microbiota, or liver enzymes. Likewise, neither NNS use nor different dietary sweet taste exposure altered sweet taste liking, appetite sensation, energy intake, or food choice. However, interpretation should consider the characteristics of the included studies, including selected populations, intervention context, outcome heterogeneity, and the fact that several behavioral and biomarker outcomes were secondary or exploratory. Overall, the reviewed evidence suggests that replacing sugar intake with NNSs may support weight management strategies, while differences in habitual dietary sweet taste exposure per se appear largely neutral with respect to health-related biomarkers and sweet taste preferences.

## 1. Introduction

Excess consumption of free and added sugars remains a major contributor to dietary energy density and the global burden of overweight, obesity, and diet-related non-communicable disease (NCD). Accordingly, dietary guidance and recommendations from global regulatory bodies and non-governmental agencies (NGO) have consistently emphasized free-sugar consumption of less than 10% of total energy intake, with additional recommendations of less than 5% for additional health benefits, as a public health priority [1]. Despite these recommendations, population-level adherence remains low [2,3].

Non-nutritive sweeteners (NNSs) provide the sweet taste of sugar but have little to no energy; they have been widely adopted as tools to reduce sugar intake while maintaining the palatability of foods and beverages. Although NNSs are among the most extensively evaluated food ingredients and are considered safe by several global regulatory authorities, their effects on weight management and cardiometabolic health have been interpreted inconsistently. This inconsistency is largely dependent on how the evidence is evaluated. Much of this controversy stems from divergent findings between randomized controlled trials (RCTs) and observational studies (OS) [4,5,6,7]. High-quality RCTs, which have appropriate controls and are designed to establish causal relationships, generally report beneficial or neutral effects of NNSs on body weight, energy intake, and glycemic outcomes, whereas observational studies, which are designed to understand correlation, frequently report adverse associations with adiposity, cardiometabolic disease, and mortality [8]. These discrepancies are widely attributed to methodological limitations between the two types of studies. RCTs investigating NNSs provide the strongest basis for causal inference because of randomization, controlled exposure, and clearly defined study objectives; however, their interpretation is often constrained by relatively small sample sizes, limited intervention duration, selected study populations, and potential influences of adherence and intervention structure, which may limit generalizability to habitual dietary patterns. In contrast, observational studies incorporate larger and more diverse populations and typically span longer timeframes, but they are susceptible to measurement error, residual confounding, and reverse causality. For instance, NNS consumption is frequently higher among individuals already exhibiting elevated metabolic risk, reducing the generalizability of associations observed in these cohorts [9].

The contrast in how the evidence is evaluated is highlighted by diverse guidelines, which typically rely more on the findings from OS: the WHO advises against the use of NNSs for weight control [8], and the recent US Dietary Guidelines for Americans (2025–2030) [10] emphasize a ‘real food’ approach and generally discourage intake of highly processed foods, including NNSs, whereas UK professional bodies (the British Dietetic Association (BDA), the British Nutrition Foundation (BNF) and Diabetes UK) recognize NNSs as suitable tools for sugar reduction [11]. Beyond questions about safety and metabolic efficacy, increasing attention has focused on sweet taste itself. Some public health guidance has extended sugar reduction messages to advocate for reducing exposure to sweetness. The WHO has stated that “people should reduce the sweetness of the diet altogether,” irrespective of whether this sweetness derives from sugars, NNSs, or naturally sweet foods [12]. The underlying rationale is that frequent exposure to sweet taste may increase liking for sweetness, thereby promoting higher consumption of sweet foods and beverages, increased energy intake, and ultimately weight gain. However, empirical evidence supporting sustained changes in sweet taste preference or long-term eating behavior as a function of sweet taste exposure remains limited, particularly from long-term controlled human studies [13].

Addressing these uncertainties and controversies regarding NNSs thus requires well-powered, rigorously designed experimental studies capable of disentangling the effects of sweetness exposure from energy intake and food form over extended periods, while evaluating the metabolic and behavioral effects of NNSs in dietary contexts reflecting habitual dietary patterns. Three large, multi-year European research consortia, SWEET (trial registration numbers: NCT04226911, NCT04633681 and NCT04483180), SWITCH (trial registration number: NCT02591134) and Sweet Tooth (trial registration number: NCT04497974), were designed to address these gaps [14,15,16,17,18,19].

This narrative review was developed in the context of the 2025 IAFNS Low- and No-Calorie Sweetener webinar series and focuses specifically on the evidence generated by the three research consortia (SWEET, SWITCH, and Sweet Tooth). These consortia were selected because they represent recent, large-scale, multi-center randomized controlled trials addressing complementary aspects of non-nutritive sweeteners and dietary sweet taste exposure. The aim of this review is, therefore, not to provide an exhaustive synthesis of all available studies, but to integrate and contextualize findings from these large, multi-center research interventions.

Collectively, these projects evaluated the safety and efficacy of NNSs under laboratory and free-living conditions, examined their role in weight loss and weight loss maintenance, assessed cardiometabolic and related biomarkers, and directly tested whether habitual exposure to sweet taste influences sweet taste preference, body weight, health-related biomarkers, or eating behavior. This narrative review synthesizes findings from these consortia to address three questions of direct public health relevance: (i) the effects of NNSs and sweet taste exposure on body weight and adiposity; (ii) their effects on cardiometabolic and related biomarkers; and (iii) their effects on sweet taste preference and eating behavior. Importantly, this review considers two related but distinct aspects: the effects of replacing added sugars with NNSs, and the effects of habitual dietary sweet taste exposure per se, irrespective of sweet taste source. By situating these findings within current guidelines and policy debates, this review aims to inform evidence-based discussion of NNS use and the role of sweet taste in the human diet.

## 2. Overview of the Included Studies

The present review synthesizes evidence from three European research consortia comprising several randomized controlled trials with different objectives and designs. Detailed study characteristics, including populations, exposures, and primary outcomes, are presented in Table 1.

**The SWEET consortium** included three randomized trials: a 1-year weight loss maintenance RCT, a mechanistic crossover trial evaluating biscuits containing NNSs, and an acute crossover beverage trial assessing postprandial metabolic responses.

The 1-year weight loss maintenance RCT (NCT04226911) examined the efficacy and safety of prolonged inclusion of NNSs within a healthy ad libitum diet during weight loss maintenance following a 2-month weight loss phase. Apart from the characteristics in Table 1, as secondary outcomes, they also included obesity-related risk factors, such as body composition (including fat mass), glucose metabolism, and lipid parameters, as well as safety-related aspects. Additional outcomes included appetite sensations, food cravings, food preferences, and sweet taste preferences [18].

The SWEET crossover biscuit trial (NCT04633681) evaluated the acute (single-day) and repeated (daily for two weeks) ingestive effects of biscuits containing NNSs on appetite-related behavioral responses and metabolic outcomes in adults with overweight or obesity. The primary aim was to assess the effects of NNSs incorporated in a solid food matrix on appetite (Table 1), while secondary outcomes included endocrine and metabolic responses following consumption, and behavioral outcomes such as food cravings and food preferences [14].

The SWEET consortium also included an acute randomized crossover beverage study (NCT04483180), designed to examine the metabolic and safety effects of beverages sweetened with different blends of NNSs compared with sucrose. The primary aim was to test whether consumption of beverages sweetened with NNS blends prior to a carbohydrate-rich meal would affect postprandial metabolic responses, particularly glycemic response markers, relative to sucrose. Secondary outcomes included appetite sensations, food intake, gastrointestinal tolerance, and initial product acceptance. Test conditions, shown in Table 1, were separated by washout periods [19].

**The SWITCH consortium** conducted a parallel-group RCT (NCT02591134) in which adults with overweight or obesity participating in a 52-week structured behavioral weight management program were randomized to consume either NNS-sweetened beverages or water. Apart from the characteristics in Table 1, secondary outcomes included measures related to body composition, appetite and eating behavior, and other biological markers associated with health [15,16].

**The Sweet Tooth consortium** included a parallel-group RCT in which overall dietary sweet taste exposure was manipulated by assigning adult participants to diets with low (10–15 E% from sweet-tasting foods), regular (25–30 E%), or high (40–45 E%) dietary sweetness exposure for six months, followed by four months of follow-up. Apart from the primary outcomes (shown in Table 1), secondary outcomes included measures of taste perception (sweet taste intensity), food choice and intake, sweet liker type, cravings, body weight, body composition, glucose variability and biomarkers related to CVD and diabetes risk. The sweetness of the overall diet was modified using both non-nutritive sweeteners and sugars, thereby altering total sensory sweet taste exposure rather than isolating a specific sweetener type. This design addressed the commonly proposed hypothesis that sustained exposure to a highly sweet diet may increase preference for sweet foods, promote higher intake of sweet products, and contribute to weight gain [17].

Not all outcomes of interest for this review were assessed across all studies, as the trials differed in their primary aims and study designs. Conversely, several outcomes measured within the individual trials were outside the scope of this review and are, therefore, not discussed here.

## 3. Effects of Non-Nutritive Sweeteners and Sweet Taste Exposure on Weight and Adiposity

All three consortia addressed the effects of NNSs or sweet taste exposure using randomized controlled designs, primarily in adults, with weight and adiposity assessed as primary or secondary outcomes. The SWEET weight loss maintenance RCT combined a low-energy diet-induced weight loss phase with randomized weight loss maintenance comparing inclusion versus avoidance of NNSs. The SWITCH study evaluated non-nutritive sweetened beverages versus water within a behavioral weight management program. The Sweet Tooth study experimentally manipulated overall dietary sweetness exposure at three distinct levels (low, regular, and high) and evaluated its effects on body weight and body composition; participants did not have weight loss goals.

**SWEET consortium**. In the 1-year weight loss maintenance RCT, both groups (including or avoiding NNSs) maintained substantial weight loss throughout a weight loss maintenance period. At 6 months, the mean body weight change was −7.5 ± 5.3 kg in the NNS avoidance group and −9.1 ± 6.0 kg in the NNS group, with a statistically significant between-group difference favoring NNS inclusion. At 12 months, both groups maintained substantial weight loss (−5.6 ± 5.7 kg vs. −7.2 ± 7.0 kg), and the group including NNS maintained significantly greater weight loss than the NNS avoidance group (between-group difference: −1.6 ± 0.7 kg). Measures of adiposity, including total fat mass, percentage body fat, visceral adipose tissue, and waist circumference, did not differ significantly between groups at 12 months, indicating similar body composition trajectories despite modest differences in weight. After 1 year, both groups maintained clinically meaningful weight loss, with the NNS group achieving 1.6 kg greater weight reduction; this was accompanied by approximately twofold greater reductions in sugar intake and stronger dietary compliance, with the highest adherence associated with the largest between-group weight difference (~3.8 kg), suggesting that consistent substitution of added sugar with NNSs may enhance weight loss maintenance [18].

**SWITCH consortium**. Following a 12-week weight management program, weight loss was equivalent between the NNS and water groups (−5.6 ± 3.0 kg for water and −5.8 ± 3.0 kg for the NNS beverage group). In a body composition subset, both fat mass and fat-free mass decreased similarly, with no between-group differences [16]. At week 52, both groups maintained significant reductions in body weight from baseline (−6.1 ± 5.8 kg with water and −7.5 ± 5.9 kg with NNS beverages), and fat mass, as well as android and gynoid fat distribution, were significantly reduced in both groups. The weight loss observed in the NNS beverage group was significantly greater than the weight loss in the water group; however, the difference did not reach the predefined 1.5 kg threshold for clinical significance [15]. These findings indicate that NNS-sweetened beverages can be incorporated within structured behavioral weight management programs supporting weight loss outcomes comparable to, or slightly better than, those observed with water.

**Sweet Tooth consortium**. Although the groups achieved distinct levels of dietary sweet taste exposure, there were no (statistically significant) changes or effects on body weight or body composition measures, including the proportion of fat mass and lean mass. Regardless of level of sweet taste exposure, body weight increased from baseline to month 7 (i.e., after completion of the 6-month intervention) by 0.5 kg and subsequently decreased between month 7 and month 10 (continued post-intervention follow-up under free-living conditions) by 0.4 kg. Likewise, fat mass increased for all groups by 0.5% between baseline and the end of the intervention (month 6) [17]. These findings indicate that higher exposure to sweet taste was associated with stable body weight and body composition.

**Figure 1:**
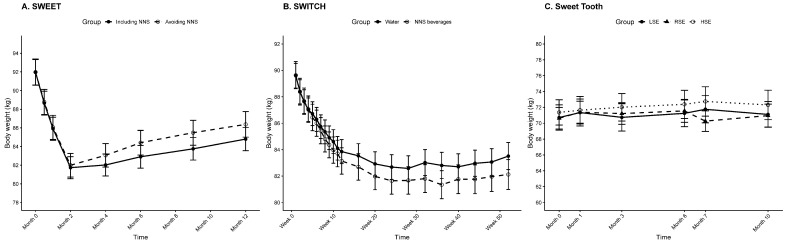
Body weight trajectories over time in the SWEET (**A**), SWITCH (**B**), and Sweet Tooth (**C**) studies. Values represent group means ± standard errors (SEs). Time reflects months for SWEET and Sweet Tooth, and weeks for SWITCH. Group labels correspond to the original trial randomizations: including NNSs vs. avoiding NNSs (SWEET; *n* = 262), Water vs. NNS beverages (SWITCH; *n* = 277), and low (LSE), regular (RSE), and high (HSE) sweetness exposure diets (Sweet Tooth; *n* = 180). SWEET and SWITCH were designed to examine the use of NNSs within weight loss maintenance interventions following initial weight loss, whereas Sweet Tooth examined the effects of differing dietary sweet taste exposure, with body weight included as an outcome rather than as a weight loss target. Figure recreated and adapted with approval from the original study authors.

Across all three consortia, evidence indicates that NNSs and dietary sweet taste exposure are associated with stable body weight and adiposity outcomes (Figure 1). In structured weight management contexts (SWEET and SWITCH), inclusion of NNSs did not impair weight loss or maintenance and was associated with modest advantages for weight control without differences in total or regional adiposity. In addition, manipulating overall dietary sweet taste exposure independent of energy restriction did not influence body weight or body composition. Collectively, these findings suggest that NNS use or higher sweet taste exposure per se does not promote weight gain and may support weight management when embedded within comprehensive dietary strategies.

## 4. Effects of Non-Nutritive Sweeteners and Sweet Taste Exposure on Biomarkers of Health

### 4.1. Glucoregulatory and Endocrine Biomarkers (Glucose, Insulin, and HbA1c; Appetite-Related Peptides)

**SWEET consortium**. In the 1-year weight loss maintenance RCT, the NNS and NNS avoidance groups did not differ in fasting glucose, insulin, or HbA1c levels at 6 or 12 months. At 6 months, fasting glucose changed by −0.17 ± 0.48 mmol/L (NNS avoidance) and −0.13 ± 0.49 mmol/L (NNS); insulin by −14.2 ± 49.6 vs. −15.4 ± 34.0 pmol/L; and HbA1c by −0.1 ± 0.2% in both groups. Likewise, changes at month 12 did not differ.

In the SWEET Biscuit crossover trial, the postprandial glucose iAUC was higher after consumption of biscuits sweetened with sucrose compared with StRebM (mean diff. of 0.09 mg/dL·min), while the difference between sucrose and neotame biscuits was not different. The insulin iAUC was also higher after sucrose biscuits compared with both StRebM (mean diff. of 0.17 μIU/mL·min) and neotame (mean diff. of 0.13 μIU/mL·min). Glucose and insulin responses were comparable on day 1 and day 14, indicating no adaptation of glycemic control after repeated exposure. Appetite-related endocrine markers, that is, ghrelin, GLP-1, and pancreatic polypeptide iAUC, did not differ between sweetener biscuit options. Pancreatic polypeptide responses were slightly higher on day 14 compared with day 1 (mean diff. of 0.22 pg/mL·min), irrespective of biscuit formulation. Overall, these findings show that replacing sucrose in solid foods with neotame or StRebM attenuates postprandial glucose and insulin responses sustainably, suggesting potential benefit for glycemic control in individuals at risk of type 2 diabetes [14].

In the SWEET crossover beverage study, detailed postprandial metabolic profiling was performed following beverage consumption (different NNS blends vs. sucrose). Compared with sucrose, all NNS blends were associated with lower 2 h glucose (mean diff. of −191.2–−354.9 mg/dL·min) and insulin (mean diff. of −1338.8–−1413.9 μIU/mL·min) iAUC responses, indicating attenuated postprandial glycemic and insulinemic responses following NNS beverages [19].

**SWITCH consortium**. At the end of the weight loss phase (week 12), fasting glucose and insulin decreased similarly in both groups. HbA1c decreased modestly in both groups, with a slightly greater, but not statistically significant, reduction in the water group (mean diff. of 0.6 mmol/L). This between-group difference was not clinically meaningful [16]. At 52 weeks, fasting glucose, insulin, and HbA1c remained stable or improved modestly from baseline in both groups, with no significant differences between water and NNS beverages [15]. These results indicate that NNS beverages supported glycemic control comparable to water during both active weight loss and weight maintenance.

**Sweet Tooth consortium**. After 6 months on diets differing in habitual sweet taste exposure, fasting glucose and insulin remained stable across groups. HbA1c decreased modestly (−0.3 mmol/L) in all groups from baseline to month 1 (i.e., during the first month of the allocated sweetness-modified intervention diets). No time × intervention effects were observed. Overall, the results indicate that modifying habitual dietary sweet taste exposure, either to a higher or lower sweet taste exposure, over 6 months, did not adversely affect glycemic control.

### 4.2. Cardiometabolic Risk Factors

**SWEET consortium**. In the 1-year weight loss maintenance RCT, no difference in blood pressure between the NNS avoidance and NNS groups was observed at 6 or 12 months. At 6 months, systolic blood pressure changed by −3 ± 11 mmHg (NNS avoidance) and −4 ± 13 mmHg (NNS), and diastolic blood pressure by −3 ± 7 and −4 ± 9 mmHg, respectively. Changes at 12 months were −5 ± 11 vs. −3 ± 10 mmHg for systolic and −3 ± 8 vs. −2 ± 7 mmHg for diastolic blood pressure. In addition, at 6 months, the NNS group exhibited greater reductions in several lipid parameters compared with the NNS avoidance group: total cholesterol (−0.31 ± 0.68 vs. −0.02 ± 0.56 mmol/L), LDL-cholesterol (−0.27 ± 0.49 vs. −0.11 ± 0.39 mmol/L), and non-HDL-cholesterol (−0.33 ± 0.66 vs. −0.10 ± 0.53 mmol/L). HDL cholesterol increased more in the NNS avoidance group (+0.08 ± 0.15 vs. +0.03 ± 0.18 mmol/L), while triglyceride reductions did not differ. At 12 months, differences were no longer statistically significant for total cholesterol, LDL cholesterol, HDL cholesterol, non-HDL cholesterol, or triglycerides. Overall, cardiometabolic trajectories were largely parallel at 12 months despite some differences at mid-intervention [18].

In the SWEET crossover beverage study, acute postprandial lipid responses showed small blend-specific effects of limited magnitude. There was an overall impact of the blend on total cholesterol, LDL cholesterol, and HDL cholesterol, but not on triglycerides. Compared with sucrose, LDL cholesterol was up to 2.9% higher, and HDL cholesterol was 1.9–2.3% lower, across the NNS blend conditions, while triglycerides did not differ, suggesting limited clinical relevance. These findings indicate that the observed lipid changes were small (<3%), below thresholds typically considered clinically relevant, and likely reflect normal biological variability rather than meaningful adverse effects of the NNS blends [19].

**SWITCH consortium**. At the end of the weight loss phase (week 12), changes in total cholesterol, LDL cholesterol, HDL cholesterol, and triglycerides were comparable between the water and NNS beverage groups, with modest improvements in both groups compared with baseline [16]. After weight maintenance (52 weeks), systolic blood pressure decreased in both groups. Similarly, lipid parameters improved modestly from baseline in both groups, with the only significant difference between groups in the changes in HDL cholesterol, driven by a modest increase from baseline in the NNS beverage group (water: 0.0 ± 0.2 vs. NNS: 0.1 ± 0.2 mmol/L) [15]. The results showed that consumption of NNS beverages within a behavioral weight management program had comparable cardiometabolic effects compared to water.

**Sweet Tooth consortium**. No effects of low, regular or high sweet taste exposure were observed on triglycerides, HDL cholesterol, or LDL cholesterol. However, an effect of sweet taste exposure was observed for total cholesterol. In the low sweet taste exposure group, total cholesterol decreased from baseline to month 1 by 0.31 mmol/L and by 0.27 mmol/L to month 3. Furthermore, irrespective of exposure group, total cholesterol decreased from baseline to month 1 by 0.16 mmol/L and remained lower at month 3 (−0.16 mmol/L) and month 7 (−0.09 mmol/L). Similarly, irrespective of exposure level, LDL cholesterol decreased from baseline to month 1 by 0.11 mmol/L and to month 3 by 0.13 mmol/L and subsequently increased between month 3 and month 10 (after the intervention) by 0.11 mmol/L. Overall, modifying the level of habitual dietary sweet taste exposure did not produce sustained or clinically meaningful changes in lipid biomarkers [17].

### 4.3. Gut Microbiota (Composition; Inferred Functional Pathways)

**SWEET consortium**. In the 1-year weight loss maintenance RCT, in a pre-specified gut microbiota subgroup, which was representative of the total population (*n* = 137), NNS inclusion was associated with distinct compositional shifts compared with the NNS avoidance group, including higher abundance of taxa linked to short-chain fatty acid (SCFA) production and methane (CH_4_) production. Inferred functional pathway analysis (PICRUSt2/MetaCyc) indicated increases in pathways related to methanogenesis and potentially saccharolytic fermentation, among other pathways, including aromatic compound degradation and l-arabinose degradation. Importantly, these microbiota changes occurred in the absence of between-group differences in T2D or CVD risk markers within the microbiota subgroup. As discussed by the authors, the observed microbial shifts were not accompanied by unfavorable changes in cardiometabolic risk markers and, therefore, did not indicate detrimental metabolic effects of NNS inclusion within the context of weight loss maintenance [18].

### 4.4. Liver Function and Broader Safety Biomarkers (ALT/AST/GGT, Intrahepatic Lipid, and Inflammatory Markers)

**SWEET consortium**. In the 1-year weight loss maintenance RCT, in the Maastricht subgroup (*n* = 27), changes in intrahepatic lipid (IHL) content during the 10-month weight loss maintenance phase did not differ between the NNS avoidance and NNS groups (−2.0 ± 3.6% vs. −2.6 ± 3.9). These findings indicate that inclusion of NNSs during weight loss maintenance was compatible with stable liver fat content. The absence of between-group differences in IHL was consistent with the overall lack of differential effects on T2D and CVD risk markers observed in the trial.

In the SWEET Crossover Beverage Trial, routine safety markers included serum alanine aminotransferase (ALT), aspartate aminotransferase (AST), and gamma-glutamyltransferase (GGT), together with calculated indices of hepatic and metabolic status (the Fatty Liver Index (FLI) and the Triglyceride–Glucose (TyG) index). Across conditions, no differences were observed between sucrose and any of the NNS blends. Liver enzymes remained within comparable ranges, and neither FLI nor TyG index values differed between interventions, indicating stable hepatic and metabolic responses following acute consumption of the tested blends [19].

**SWITCH consortium**. At the end of the weight loss phase (12 weeks), liver enzymes (ALT, AST and GGT) remained stable or improved modestly in both groups, with no differences between groups [16]. Similarly, at 52 weeks, liver enzymes remained stable in both groups, without meaningful between-group differences. These results indicate that consumption of NNS beverages does not affect liver function markers compared with water.

Across the three consortia, evidence indicates that NNS consumption and dietary sweetness exposure are compatible with stable biomarkers of cardiometabolic and related health. In the SWEET trials, replacing sucrose with NNSs in both acute and longer-term interventions produced comparable or more favorable postprandial glucose and insulin responses, while fasting cardiometabolic markers, including blood pressure, lipids, liver enzymes, and intrahepatic lipids, remained similar between NNS and NNS avoidance conditions. Although small shifts in gut microbiota composition were observed in the SWEET sub-study, these changes were modest in magnitude and not accompanied by differences in established cardiometabolic risk markers. In the SWITCH trial, consumption of NNS beverages within a structured behavioral weight management program resulted in glycemic, lipid, blood pressure, and liver enzyme trajectories comparable to those observed with water across both the weight loss and weight maintenance phases. Similarly, in the Sweet Tooth trial, modifying habitual dietary sweet taste exposure over six months did not affect fasting glucose, insulin, HbA1c, lipid markers, or other cardiometabolic indicators. Collectively, these findings suggest that the use of NNSs, or higher dietary sweet taste exposure per se, can be incorporated into typical dietary patterns while maintaining stable glycemic control, cardiometabolic risk markers, liver function, and related biomarkers, and may support dietary strategies aimed at reducing sugar intake.

## 5. Effects of Non-Nutritive Sweeteners and Sweet Taste Exposure on Sweet Taste Preference and Eating Behaviors

### 5.1. Sweet Taste Liking and Preference

**SWEET consortium**. In the 1-year weight loss maintenance RCT, liking for sweet foods was assessed by the Leeds Food Preference Questionnaire (LFPQ) [20]. The LFPQ compares appeal for high-fat and low-fat foods (fat appeal) and sweet and savory foods (sweet appeal) using validated photographic food stimuli. The results showed that inclusion of NNSs did not increase explicit liking nor implicit wanting for sweet foods, while avoidance of NNSs led to an increase in explicit liking toward sweet (sugar-containing) foods. These findings do not support the hypothesis that intake of NNSs reinforces a preference for sweet taste. On the contrary, avoidance of NNSs during the 1-year weight maintenance period led to a greater liking of sweet foods [21,22].

In the SWEET Crossover Biscuit Trial, sweet food preference was also assessed using the LFPQ [20]. Across both the acute exposure (day 1) and after 14 days of daily consumption of biscuits sweetened with sucrose, neotame, or StRebM, no significant differences were observed in explicit liking or implicit wanting for sweet foods between sweetener conditions. Furthermore, repeated exposure over 2 weeks did not modify sweet preference measures relative to sucrose, indicating no sensitization or attenuation effects on food reward [14]. Thus, replacing sucrose with neotame or StRebM in a solid food matrix did not alter sweet liking or wanting, either acutely or following repeated consumption, providing no evidence that NNS exposure reinforces sweet food preference in the short term.

In the SWEET Crossover Beverage Trial, acute liking and desire for test beverages were assessed immediately after consumption using 100 mm visual analog scales (VAS). This measure reflected short-term sensory acceptance of beverage formulations rather than habitual sweet preference. Significant differences in liking and desire ratings were observed across different blends. Sucrose and a sucralose–acesulfame-K blend were rated as more liked and more desired than both stevia-containing blends [19]. Thus, the trial demonstrated formulation-dependent differences in acute hedonic acceptance, without assessing longer-term changes in sweet taste liking or preference.

**Sweet Tooth consortium.** In the 6-month parallel RCT, sweet taste liking was assessed in actual food products varying in sweetness concentration (ranging from not sweet at all to extremely sweet) [23]. Participants tasted the samples and rated both liking and perceived sweetness intensity under controlled conditions. No effect of low, regular or high dietary sweet taste exposure manipulation was observed on sweet preference, sweet taste liking or sweetness intensity perception [17]. Overall, modifying habitual dietary sweet taste exposure for 6 months did not alter sweet taste preference or perceptual sensitivity of sweet taste.

### 5.2. Appetite, Cravings and Food Intake

**SWEET consortium**. In the 1-year weight loss maintenance RCT, appetite sensations and eating behavior were assessed using validated self-report instruments, including VAS for hunger and satiety and the Control of Eating Questionnaire (CoEQ) for craving and appetite control [24]. Inclusion of NNSs during weight maintenance was not associated with increased hunger or reduced satiety compared with the NNS avoidance group. Sweet cravings were lower in the NNS group at 6 months, and overall appetite regulation did not differ between groups across the 12-month period [18]. In a sub-study nested within the same RCT (*n* = 26), acute appetite responses were assessed during controlled laboratory test days at baseline, after the 2-month weight loss phase, and after a 4-month weight loss maintenance period. Participants consumed a standardized breakfast followed by a test drink containing a mixture of acesulfame-K and cyclamate or water, and appetite sensations were repeatedly measured using a VAS during a 4 h postprandial period. An ad libitum pizza test meal was subsequently provided to assess actual food intake. The results showed that the NNS condition was associated with lower ratings of hunger, prospective consumption, and desire to eat something sweet compared with the NNS-avoiding group consuming water. However, hunger differences were attenuated after adjusting for the taste of the test drinks. Importantly, ad libitum energy intake at the test meal did not differ between conditions, and repeated exposure to NNSs during weight loss and weight maintenance did not modify the initial appetite responses or ad libitum intake over time [25]. These findings together indicate that inclusion of NNSs within a structured weight management program supports stable appetite control and may reduce prospective consumption and desire for something sweet.

In the SWEET Crossover Biscuit Trial, appetite sensations were also measured using a VAS. No significant differences were observed in hunger, fullness, or prospective food consumption between sweetener conditions, either acutely or after repeated exposure (14 days). Thus, replacing sucrose with neotame or StRebM in a solid food matrix did not alter short-term appetite regulation [14].

In the SWEET Crossover Beverage Trial, acute appetite sensations were measured using repeated VAS ratings following consumption of sucrose- or NNS-sweetened beverages under standardized conditions. In addition, ad libitum food intake was assessed over the subsequent 24 h. Although minor blend-specific differences were observed in fullness and prospective consumption, these effects were small and did not translate into differences in total 24 h energy intake between conditions [19]. Overall, NNS-sweetened beverages were associated with energy intake comparable to sucrose over 24 h.

**SWITCH consortium**. In the 12-week weight loss phase and 52-week weight maintenance phase, hunger sensations were assessed using VAS ratings, while habitual intake of sugars and sweeteners was assessed using the Sugar and Sweetener Food Frequency Questionnaire (SSFFQ) [16]. Reductions in hunger during active weight loss were comparable between the NNS beverage and water groups. Likewise, during weight maintenance, no differences were observed between groups in hunger measures. During both the weight loss and weight maintenance phases, sugar consumption decreased from baseline in both the water and NNS groups and did not differ significantly between groups. However, the reduction in sweetener consumption was greater in the water group compared with the NNS group, both during weight loss (−13.5 vs. −0.7 score points) and weight maintenance (−13.1 vs. +1.2 score points). Thus, the SSFFQ confirmed good intervention adherence [15,16]. These findings indicate that NNS beverage consumption did not increase hunger compared with water in either short- or long-term settings.

**Sweet Tooth consortium**. In the 6-month parallel RCT, food intake was assessed both in a controlled laboratory setting and by self-report. In the laboratory assessments, food choice and energy intake were measured during standardized ad libitum breakfast buffet meals, where participants were offered a variety of foods differing in taste modalities (sweet, savory, neutral, fatty, and bitter) and could eat until pleasantly satisfied. The proportion of sweet versus non-sweet foods consumed, as well as total energy and proportion of sweet foods, was calculated from covertly weighed food intake. The results showed no differences in total energy intake between the low, regular, and high dietary sweet taste exposure groups. Similarly, the proportion of sweet foods chosen at breakfast meals did not differ between groups, indicating that higher habitual exposure to sweet-tasting foods was associated with similar consumption of sweet foods in the controlled test setting. Habitual dietary intake was assessed using repeated web-based 24 h dietary recalls, in which participants reported all foods and beverages consumed the previous day [26]. Self-reported daily energy intake showed differences only early in the intervention: at month 1, the low sweet taste exposure group reported lower energy intake compared with the regular sweet taste exposure group (mean difference: 311 kcal). This difference was not sustained at later time points, and no between-group differences were observed at other time points. Although sweet food intake differed between groups during the intervention, as expected from the dietary manipulation, these differences were not maintained after the intervention ended. During the follow-up period, participants in both the low and high sweet taste exposure groups returned to levels of sweet food consumption similar to regular, baseline intakes [17]. Overall, these findings indicate that varying dietary exposure to sweet-tasting foods does not influence sweet food intake or total energy intake, and any intervention-induced changes in sweet taste consumption dissipated once the manipulation ended.

Across the three consortia, there was little evidence that NNS use or greater habitual exposure to sweet taste increased sweet preference, appetite, or subsequent food intake. In SWEET, inclusion of NNSs during weight loss maintenance did not increase explicit liking or implicit wanting for sweet foods, whereas avoidance of NNSs was associated with greater liking for sweet foods over time. In the SWEET biscuit trial, replacing sucrose with neotame or StRebM did not alter sweet liking or wanting, either acutely or after repeated exposure. In the SWEET beverage trial, short-term differences in liking reflected formulation-specific sensory acceptance rather than changes in habitual sweet preference. Similarly, in Sweet Tooth, six months of low, regular, or high dietary sweet taste exposure did not modify sweet taste liking or sweet preference. Appetite ratings, cravings, and energy intake were also broadly comparable across interventions, with only minor short-term differences that did not translate into consistent differences in ad libitum intake. Finally, in the SWITCH trial, replacing water with NNS beverages within a behavioral weight management program did not increase hunger. Overall, these findings do not support the view that exposure to NNSs, or to a sweeter diet per se, reinforces sweet preference or dysregulates appetite.

## 6. Conclusions

Across the SWEET, SWITCH, and Sweet Tooth consortia, the evidence was broadly consistent in showing no consistent evidence of adverse effects of NNS use or differences in habitual dietary sweet taste exposure on body weight, health-related biomarkers, sweet taste preference, or eating behavior (Table 2).

Replacing sugars with NNS-containing foods or beverages was associated with improved weight loss maintenance, favorable body weight and glucoregulatory outcomes, while cardiometabolic markers remained stable (SWEET; SWITCH) [14,15,16,18,19,27]. These findings are consistent with systematic reviews and meta-analyses of randomized controlled trials indicating that replacing sugars with NNSs reduces energy intake, contributes to modest weight loss over time, and does not affect cardiometabolic markers [9,28,29,30,31,32,33,34,35,36].

The trials reviewed here also addressed prevailing concerns that exposure to sweet taste, including from NNSs, may increase appetite or reinforce preference for sweet foods. Across SWEET and Sweet Tooth, sweet taste liking, preference, food intake and appetite remained largely stable despite differences in dietary sweet taste or NNS exposure. This interpretation is further supported by reviews indicating that effects of sweeteners and sweetness enhancers on appetite and food reward are not consistently accompanied by increased food intake or adverse metabolic/adiposity outcomes in adults [34,37,38]. The results are further consistent with systematic reviews indicating that long-term sweetness exposure per se does not alter sweet taste preference and appetite [13,31,39].

The individual trials included in this review have some limitations. Although RCTs provide the strongest basis for causal inference, the studies reviewed here were conducted in selected European populations, mainly adults, with overweight or obesity, often within structured dietary or behavioral intervention contexts. Accordingly, the findings may not fully generalize to other population groups or habitual real-world dietary patterns. Some outcomes were secondary or exploratory, and power may have been limited for mechanistic endpoints, subgroup analyses, and longer-term follow-up. In addition, higher-than-expected dropout rates and potential underreporting of dietary intake should be considered when interpreting the findings. Null findings should, therefore, be interpreted as an absence of detectable differences within the conditions, populations, and timeframes studied, rather than definitive evidence of no effect. At the review level, interpretation is further constrained by the targeted inclusion of studies from only three consortia.

However, when considered together, the SWEET, SWITCH, and Sweet Tooth provide complementary evidence across different populations, intervention types, and experimental approaches. Taken together, these findings are consistent with the existing body of evidence that replacing sugars with NNSs can support weight management strategies and reduction in sugar intake, whereas variation in habitual dietary sweet taste exposure per se (independent of whether sweetness is derived from sugars or NNSs), within the studied intervention periods, appears largely neutral with respect to weight regulation, health-related biomarkers, and eating behavior. However, long-term effects and developmental effects, particularly in children and adolescents, remain uncertain.

## Figures and Tables

**Table 1:** Overview of the trials included in this review, summarizing study design, participant characteristics, exposure conditions, duration, and the primary outcomes assessed in each study.

Study (Year)	Design	Participants	Exposure	Duration	Primary Outcome(s)
SWEET weight maintenance trial (2020–2022)	Parallel RCT	341 adults with overweight/obesity	NNS inclusion vs. avoidance within a healthy diet	12 months (2 months weight loss + 10 months weight maintenance)	Body weight change; gut microbiota change
SWEET biscuit trial (2021–2022)	Crossover	53 adults with overweight/obesity	Biscuits with sucrose vs. neotame vs. steviol glycoside rebaudioside M	2 weeks per condition	Appetite sensations
SWEET beverage trial (2020–2021)	Crossover	59 adults with overweight/obesity	NNS beverage blends vs. sucrose	Acute	Postprandial metabolic responses (glycemic markers)
SWITCH (2016–2024)	Parallel RCT	493 adults with overweight/obesity	NNS beverages vs. water	52 weeks (12-week weight loss phase; 40-week weight maintenance phase)	Body weight change
Sweet Tooth (2020–2024)	Parallel RCT	180 adults; normal weight/with overweight	Low vs. regular vs. high dietary sweetness exposure	6 months + 4-month follow-up	Preference changes for sweet foods and beverages

**Table 2:** Summary of evidence from the SWEET, SWITCH, and Sweet Tooth consortia on non-nutritive sweeteners (NNSs) and dietary sweet taste exposure.

Trial	Exposure	Body Weight	Biomarkers of Health	Sweet Taste Preference and Eating Behavior
SWEET weight maintenance	NNS inclusion vs. avoidance within a healthy diet	★	○	★
SWEET biscuit	Biscuits with sucrose vs. neotame vs. RebM	–	★	○
SWEET beverage	NNS beverage blends vs. sucrose	–	★	○
SWITCH	NNS beverages vs. water	★	○	○
Sweet Tooth	Low vs. regular vs. high dietary sweet taste exposure	○	○	○

Note: Symbols in the table provide qualitative summary of the direction of evidence only and should not be interpreted as indicating the magnitude of effects. Symbols: ★ evidence of a favorable between-condition difference for the specified outcome domain, based on the primary interpretation reported in the corresponding trial; ○ no detectable difference between conditions; – outcome not assessed in this study. Abbreviations: NNS, non-nutritive sweetener.

## Data Availability

No new data were created or analyzed in this study. Data sharing is not applicable to this article.

## Abbreviations

The following abbreviations are used in this manuscript:

ALT	Alanine aminotransferase
AST	Aspartate aminotransferase
CoEQ	Control of Eating Questionnaire
CVD	Cardiovascular disease
E%	Percentage of total energy intake
FLI	Fatty Liver Index
GGT	Gamma-glutamyltransferase
GLP-1	Glucagon-like peptide-1
HbA1c	Glycated hemoglobin
HDL	High-density lipoprotein cholesterol
iAUC	Incremental area under the curve
IHL	Intrahepatic lipid
LDL	Low-density lipoprotein cholesterol
LFPQ	Leeds Food Preference Questionnaire
NCD	Non-communicable disease
NNS	Non-nutritive sweetener
OS	Observational studies
RCT	Randomized controlled trial
SSFFQ	Sugar and Sweetener Food Frequency Questionnaire
StRebM	Steviol glycoside rebaudioside M
T2D	Type 2 diabetes
TyG	Triglyceride–Glucose index
UK	United Kingdom
US	United States
VAS	Visual analog scale
WHO	World Health Organization
